# Ultra-Rapid Cycling Bipolar Disorder: A Case Report

**DOI:** 10.7759/cureus.115491

**Published:** 2026-08-31

**Authors:** Shaheen K Kaplan, Jawad Zafar, Kayla Lovejoy

**Affiliations:** 1 Psychiatry and Behavioral Sciences, Avalon University School of Medicine, Willemstad, CUW; 2 Department of Behavioral Medicine and Psychiatry, Farhat Medical Clinic, Beckley, USA

**Keywords:** bipolar disorder (bpd), electroconvulsive therapy, generalized anxiety disorder-7, patient health questionnaire-9, ultra-rapid cycling bipolar disorder

## Abstract

Ultra-rapid cycling disorder, in which mood episodes shift over days rather than months, sits at one of the most difficult ends of the bipolar spectrum. It resists standard treatment, tends to worsen with antidepressants, and has very little long-term outcome data to guide clinical decisions.

We report the case of a 51-year-old woman whose ultra-rapid cycling bipolar disorder was tracked for 130 weeks. She underwent trials of multiple mood stabilizers, five second-generation antipsychotics, and two courses of electroconvulsive therapy (ECT), none of which produced sustained remission. Clozapine brought the most meaningful improvement seen across the entire treatment period but had to be stopped because of sialorrhea and weight gain. The patient described the sialorrhea as feeling like she was drowning in her own saliva, a symptom that, while pharmacologically manageable, was not addressed in time to preserve the treatment. She was subsequently transitioned to long-acting injectable aripiprazole, primarily to address adherence.

Ultra-rapid cycling bipolar disorder is genuinely hard to treat, and this case shows what that looks like in practice. Sustained remission may require earlier procedural intervention, more aggressive management of comorbidities, and a willingness to consider agents, including clozapine, that are often reserved as a last resort.

## Introduction

Rapid cycling bipolar disorder, defined as four or more mood episodes within 12 months, already carries a worse prognosis than the noncycling form of the illness. Ultra-rapid cycling takes this further. Episodes here shift over days to weeks, sometimes within the same week, leaving patients and clinicians with little time to establish whether a treatment is working before the clinical picture has already changed again [1].

The condition is not formally codified in the Diagnostic and Statistical Manual of Mental Disorders, Fifth Edition (DSM-5) as a separate specifier, but it is recognized in the clinical literature as a distinct and particularly refractory phenotype [1]. Epidemiological data are limited, partly because the rapid shifts make standardized assessment difficult. What is clear is that this group tends to accumulate treatment failures, have higher rates of hospitalization, and experience significant functional deterioration over time. The human cost of this is not abstract: lifetime rates of suicide attempts in bipolar disorder overall approach 25% to 35%, and within the rapid-cycling subgroup, that burden is even higher [2]. These are not statistics that sit in the background when you are sitting across from a patient who has attempted suicide more than 10 times. They are the context in which every prescribing decision is made.

Treatment is constrained from multiple directions. Antidepressants, which might seem an obvious tool given the depressive loading, can accelerate cycling and are generally contraindicated [3]. Lithium and valproate remain the backbone of bipolar pharmacotherapy, but tolerability issues and incomplete responses are common [4,5]. Lamotrigine offers some benefit for the depressive phase of bipolar disorder but has not been shown to reliably prevent manic or rapid-cycling episodes [6]. Second-generation antipsychotics are often added or substituted, though the evidence base for their use specifically in ultra-rapid cycling is thin, and systematic reviews have not identified any single agent as clearly superior in this subgroup [7]. Electroconvulsive therapy (ECT) has been used in refractory cases and can be delivered as an acute or maintenance intervention, but it remains underutilized and understudied in this population [8,9].

This case documents the longitudinal treatment course of one such patient over two and a half years. We present it not as a success story, as she remains unwell, but because the clinical granularity of her trajectory, captured through serial Patient Health Questionnaire-9 (PHQ-9) and Generalized Anxiety Disorder-7 (GAD-7) assessments, offers something the broader literature lacks: an honest, close-grained account of what managing this condition actually involves.

## Case presentation

The patient is a 51-year-old woman with a diagnosis of ultra-rapid cycling bipolar disorder, first identified in 2018, two years after an initial bipolar disorder diagnosis. Her history includes more than 10 suicide attempts by overdose, a number that is difficult to sit with and one that reflects both the severity of her illness and the years during which it went inadequately managed [2]. She also has intermittent auditory hallucinations and alcohol use disorder, two comorbidities that have each independently complicated her care and, in combination, created a clinical picture that has resisted straightforward management. Psychotic features occur in a significant proportion of patients with bipolar disorder and are associated with greater illness severity, more frequent hospitalizations, and poorer treatment outcomes. In her case, the psychotic dimension was present earlier in the clinical picture than the treatment plan fully acknowledged, and it is reasonable to wonder whether earlier recognition of this feature would have changed the sequencing of interventions.

Longitudinal symptom tracking using the PHQ-9 and GAD-7 was conducted at regular outpatient visits between week 1 and week 130, as shown in Figure 1. Both instruments are validated, widely used, and critically sensitive to change over time, which makes them suited to tracking treatment response even when they were not originally designed for bipolar disorder [10,11]. PHQ-9 scores over this period ranged from 9 to 24, and GAD-7 scores from 6 to 26. The variability of these scores, shifting substantially from visit to visit, is itself a picture of the disorder (Figure 1).

**Figure 1:**
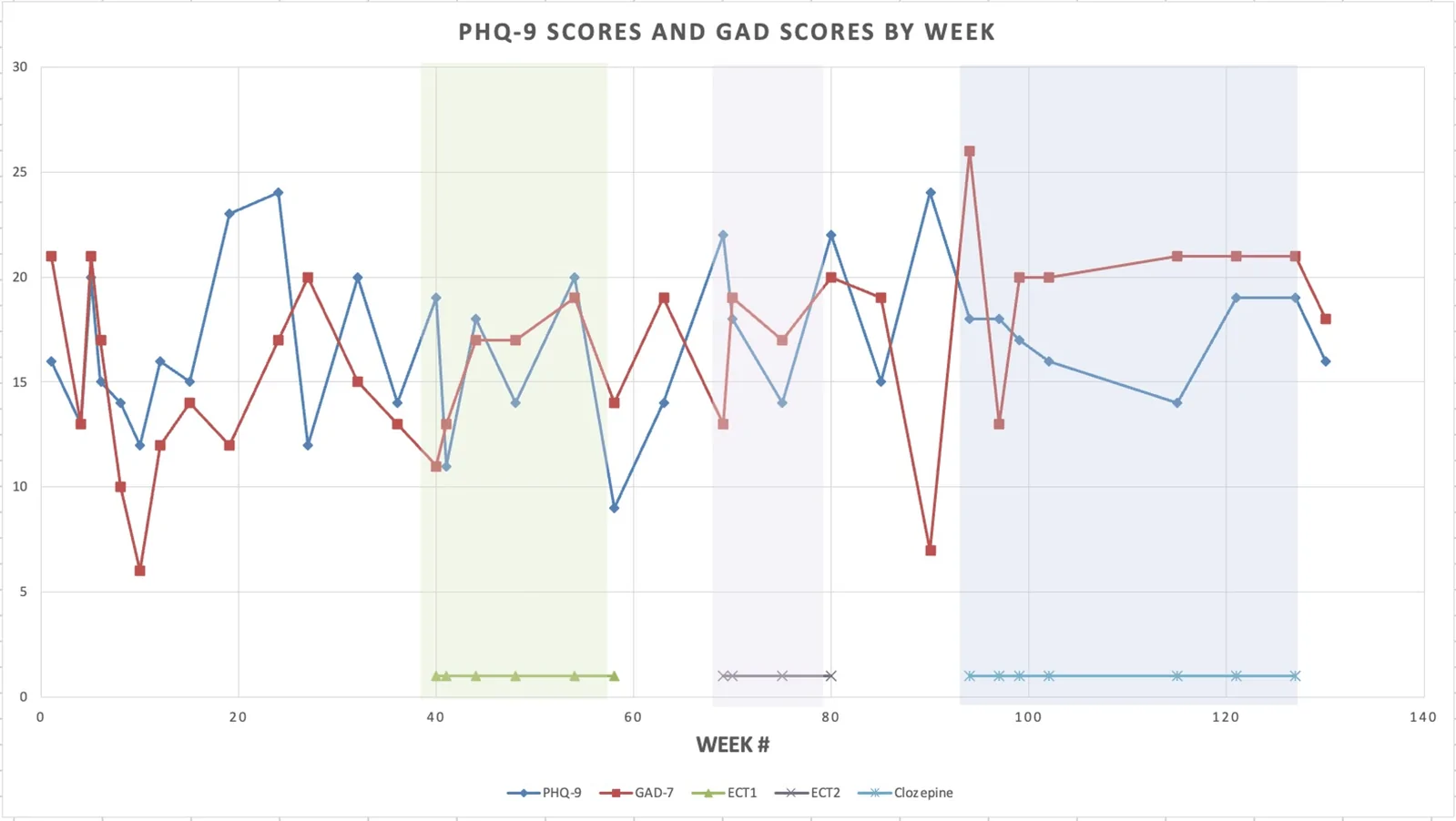
PHQ-9 and GAD-7 scores by week PHQ-9, Patient Health Questionnaire-9; GAD-7, Generalized Anxiety Disorder-7; ECT1, 1st electroconvulsive therapy treatment; ECT2, 2nd electroconvulsive therapy treatment

Prior mood stabilizers

Before the period under review, the patient had been prescribed divalproex sodium and lamotrigine. Divalproex was stopped after she developed weight gain and elevated liver enzymes, a risk compounded by her alcohol use disorder, since hepatic stress from both sources is not additive but potentially synergistic [5]. Lamotrigine was trialed but was not continued. Its evidence base in bipolar disorder is strongest for depressive relapse prevention, and it has not consistently demonstrated benefit in patients with the rapid-switching pattern [6]. Lithium was introduced in week 1, titrated carefully, and has since been maintained at 600 mg each morning and 300 mg at bedtime [4]. It is one of the few pharmacologic decisions in this case that has held.

Antipsychotic trials

Five second-generation antipsychotics were tried in sequence: olanzapine, quetiapine, lurasidone, lumateperone, and cariprazine. Each was started with a reasonable clinical rationale, and each was eventually stopped, some because they did not work and others because the side effects were unacceptable to the patient. Weight gain and sedation were recurring problems. The serial failure of this drug class in her case reflects both the biology of ultra-rapid cycling and the limited evidence base for any single agent in this phenotype [1].

Electroconvulsive therapy

ECT was initiated in week 40 after pharmacotherapy had failed to produce sustained improvement. Following the induction of general anesthesia with methohexital and neuromuscular blockade with succinylcholine, the patient was preoxygenated, and a bite block was placed to ensure airway and dental protection. A controlled, constant-current electrical stimulus was applied bitemporally via scalp electrodes at a pulse frequency of 60 Hz for a predefined train duration of 2.0 seconds to trigger a generalized therapeutic seizure. Brain wave activity and peripheral motor convulsions were continuously tracked, and once the generalized seizure had fully resolved on the electroencephalogram (EEG), the patient was transitioned to the postanesthesia care unit for monitoring and recovery. Two separate courses were completed. Both produced partial responses, reflected by PHQ-9 and GAD-7 data during the treatment windows (Figure 1). ECT was not continued beyond the second course; logistical barriers and the patient’s difficulty recovering after sessions made it unsustainable in her particular circumstances. The partial responses observed are nonetheless clinically meaningful and suggest that procedural intervention has a role in her treatment picture, even if that role could not be fully realized [8].

Clozapine

Clozapine was started in week 94 and titrated to 300 mg daily. Across the treatment period documented here, it produced the most substantial improvement. Both depressive symptoms and intermittent auditory hallucinations, a psychotic feature that had been present but underweighted in earlier treatment planning, responded meaningfully [10]. PHQ-9 and GAD-7 scores during the clozapine period showed a sustained downward trend not seen at any other point in the chart (Figure 1). Clozapine was discontinued in week 127 after the patient developed sialorrhea and gained significant weight, both of which she found intolerable. The sialorrhea, in particular, was described as a profound clinical challenge: she told her nurse practitioner that she felt like she was drowning in her own saliva sometimes. That is not a clinical abstraction; it is a visceral, daily experience that made the medication impossible to live with, regardless of how well it was working. Sialorrhea (excessive drooling) is one of the most commonly reported and most practically disruptive side effects of clozapine, and it is also one of the most pharmacologically addressable: anticholinergic agents such as glycopyrrolate or hyoscine have demonstrated meaningful reductions in clozapine-induced hypersalivation in clinical trials [10]. Weight gain, which can be substantial with clozapine, is similarly not unmanageable: structured dietary support, exercise programs, and adjunctive metformin have each shown benefit in controlled studies [10]. Whether these strategies would have allowed her to continue on clozapine is unknown. Still, the observation is worth noting explicitly: the most effective agent she encountered across a two-and-a-half-year treatment course was stopped for side effects that have established mitigation pathways. That is a clinical and systemic gap.

Current regimen

Following clozapine, oral aripiprazole 30 mg daily was started in week 127 and converted to a long-acting injectable formulation at 400 mg monthly [10]. The primary rationale was adherence rather than evidence of superior efficacy. Her current regimen is lithium 600 mg each morning and 300 mg at bedtime; aripiprazole long-acting injectable 400 mg monthly; buspirone 20 mg three times daily; and hydroxyzine 25 mg twice daily as needed for anxiety. Duloxetine was tapered and discontinued. A short course of doxepin 25-50 mg at bedtime has also been prescribed for insomnia. Alcohol use disorder has not been formally addressed with pharmacotherapy or structured psychosocial intervention during the period under review. The evidence supports integrated, simultaneous treatment of both conditions rather than sequential approaches, which tend to leave each comorbidity underserved [10]. Incorporating naltrexone or acamprosate alongside ongoing mood stabilization is a reasonable next step in her care planning.

She continues to experience ultra-rapid cycling mood episodes and intermittent auditory hallucinations. Remission has not been achieved.

## Discussion

What this case illustrates, perhaps more than anything else, is that ultra-rapid cycling bipolar disorder does not yield to persistence alone. This patient received serious, sustained psychiatric care across two and a half years. Multiple pharmacological strategies were tried in good faith. ECT was initiated, yet she remains symptomatic. That outcome is not a clinician’s failure, but it reflects the biology of a condition that our current therapeutic toolkit does not yet reliably address [1].

The serial failure of five second-generation antipsychotics is worth dwelling on. In clinical practice, these agents are often cycled through with the implicit assumption that one will eventually work. In ultra-rapid cycling bipolar disorder, that assumption may not hold. The cycling mechanism in this phenotype may be neurobiologically distinct from that of classical bipolar disorder, and the relative absence of randomized controlled trial data specifically for this group means that most prescribing decisions are based on extrapolation [1]. At some point, continuing to try agents from a class that has repeatedly failed is not persistence but the path of least resistance.

The comorbid alcohol use disorder was not a peripheral finding in this case; it was a recurring structural problem. It contributed to hepatotoxicity, which ruled out valproate [5]. It destabilized her mood cycling independently, and it may have blunted the effectiveness of agents that otherwise might have helped [10]. Managing alcohol use disorder in parallel with bipolar disorder is not optional when the two conditions are this closely intertwined. Sequential treatment, addressing the mood first, then the alcohol, or vice versa, is an approach that will consistently fall short in this kind of complex comorbidity [10]. Integrated dual-diagnosis treatment models, which address both conditions within the same care framework, have demonstrated superior outcomes to sequential approaches and are increasingly endorsed in the literature, yet remain difficult to access in community psychiatric settings [10]. This patient’s alcohol use has not been formally treated with pharmacotherapy or structured psychosocial intervention during the period reviewed here. That gap is clinically significant.

The ECT story in this case is one of constrained access rather than failed efficacy. Both courses produced partial improvement. The patient did respond. What could not be sustained were the logistics: recovery time, travel, and scheduling. These are not trivial barriers, particularly for someone with the functional impairment that accompanies ultra-rapid cycling. Maintenance ECT, less frequent but sustained over months, is an established option for treatment-resistant mood disorders [8], with protocols delivering monthly or bimonthly sessions shown to preserve response in patients who initially benefited from acute courses [9]. There is a reasonable case that this option should have been explored more explicitly here, not as a replacement for pharmacotherapy, but as a parallel strategy that might have extended the partial gains observed during each acute course. The broader systemic point is that ECT remains difficult to access in community and rural psychiatric settings, and that difficulty falls hardest on exactly the patients who need it most.

Clozapine’s response in this case is consistent with a growing body of evidence supporting its use in treatment-resistant bipolar disorder, particularly where psychotic features are present [10]. What made this case distinctive was the dual outcomes: a mood-stabilizing effect and a reduction in auditory hallucinations, both reflected in objective scoring. The decision to discontinue it was clinically defensible, as sialorrhea and significant weight gain are real and burdensome side effects, but it is worth noting that clozapine is often abandoned prematurely before tolerability management strategies have been genuinely exhausted. In her case, the sialorrhea was not a mild inconvenience. She described feeling like she was drowning in her own saliva, a phrase that conveys, more precisely than any rating scale, why she could not continue. And yet the pharmacological tools to address this symptom exist and are well-evidenced. Glycopyrrolate or hyoscine for sialorrhea, administered sublingually or orally, are well-evidenced and relatively well-tolerated interventions for this specific problem [10]. A structured weight management program, dietary counseling, physical activity support, and consideration of adjunctive metformin represent a parallel effort that has demonstrated benefit in patients on clozapine and other metabolically burdensome antipsychotics [10]. These are not minor add-ons; they are the difference between a patient being able to stay on the most effective agent she had found and losing it entirely. When a patient uses language like “drowning in her saliva,” that is a signal not just to stop the drug but to ask whether the symptom can be treated alongside it. That question, in this case, was not asked in time, as it is often difficult to predict which symptoms will be most concerning to the patient. The clinical imperative, where clozapine is working, is to invest in tolerability management before accepting discontinuation.

The transition to long-acting injectable aripiprazole reflects a pragmatic and reasonable clinical decision. In this patient, the history of multiple suicide attempts and complex comorbidities created real adherence risks, making the removal of the daily pill burden meaningful [10]. Whether aripiprazole will provide the mood stabilization that other antipsychotics did not is uncertain. Its evidence base in rapid-cycling bipolar disorder exists but is modest, and its metabolic profile, at least, is more favorable than those of most alternatives she has tried.

The use of serial PHQ-9 and GAD-7 scores as the backbone of longitudinal tracking in this case deserves comment. The PHQ-9 was originally validated as a screening and severity instrument for major depressive disorder, and the GAD-7 for generalized anxiety disorder; neither was designed with bipolar disorder in mind [10,11]. They do not capture manic or hypomanic episodes directly, and a patient who is acutely manic may score low on the PHQ-9 despite being clinically unwell. That is a genuine limitation. What they do provide, when administered consistently over time, is a quantitative record of symptom trajectory that makes temporal associations between treatment changes and clinical outcomes visible in a way that narrative case notes do not. The data in Figure 1 tell a story: the relative stability during lithium initiation, the partial responses during ECT, the most sustained improvement during clozapine, and the renewed instability after its discontinuation. That story is clinically useful and methodologically reproducible, and it is the kind of longitudinal signal that is very hard to reconstruct retrospectively from clinical notes alone. More widespread adoption of this kind of systematic outpatient monitoring would benefit both individual patients and the cumulative evidence base for treatment-resistant bipolar disorder.

There is no clean resolution here. The patient remains in treatment, the regimen is still being adjusted, and sustained remission has not been achieved. What this case offers instead is an unvarnished account of what treating ultra-rapid cycling bipolar disorder looks like when the standard playbook runs out, and what decisions face clinicians when it does. The lessons that emerge, about tolerability management, comorbidity integration, and the real-world constraints on procedural treatment, are not unique to this patient. They are features of the illness and the system in which it is treated.

Limitations

This report has limitations that should be acknowledged. First, it is a single case. The treatment decisions and outcomes documented here cannot be generalized to the broader ultra-rapid cycling population, and it would be a mistake to read any individual finding, such as the clozapine response, the partial improvement with ECT, or the failure of five antipsychotics, as more than a clinical observation from one patient’s course. Second, the PHQ-9 and GAD-7 were used as the primary outcome instruments throughout. While their serial use provides a quantitative longitudinal record, they do not capture hypomanic or manic episodes, and they were not supplemented with a validated bipolar-specific instrument such as the Young Mania Rating Scale (YMRS) [12] or the Bipolar Depression Rating Scale (BDRS) [13]. The picture they provide is real but not complete. Third, no formal neuropsychological assessment or brain imaging was conducted during the treatment period, which means that potential structural or neurobiological contributors to her refractory course remain uncharacterized. Fourth, alcohol use was tracked clinically rather than through structured instruments or biomarkers, and its real impact on treatment response across the review period is difficult to quantify precisely. These limitations do not undermine the value of the longitudinal clinical record, but they are relevant to how it is interpreted.

## Conclusions

Ultra-rapid cycling bipolar disorder remains difficult to treat when standard interventions fail. This case highlights the value of proactive tolerability management for effective agents, integrated comorbidity treatment, and systematic longitudinal monitoring. Earlier consideration of clozapine and maintenance ECT may improve outcomes in refractory cases.
